# Supplementary material for: Mammalian pumilio proteins control cellular morphology, migration, and adhesion
Source: Sci Rep. 2023 Feb 21;13:3002. doi: 10.1038/s41598-023-30004-4 (PMC9944931; doi:10.1038/s41598-023-30004-4)
Supplement: Supplementary file 4 — Supplementary Information 5. [file 41598_2023_30004_MOESM4_ESM.docx]

### Supplementary Figures and Supplementary Video Legends for:

# Mammalian Pumilio Proteins Control Cellular Morphology, Migration, and Adhesion

Erin L. Sternburg, Jordan J. Lillibridge, Rattapol Phandthong, and Fedor V. Karginov*

Department of Molecular, Cell and Systems Biology, University of California at Riverside, Riverside, CA 92521

* Corresponding author: karginov@ucr.edu


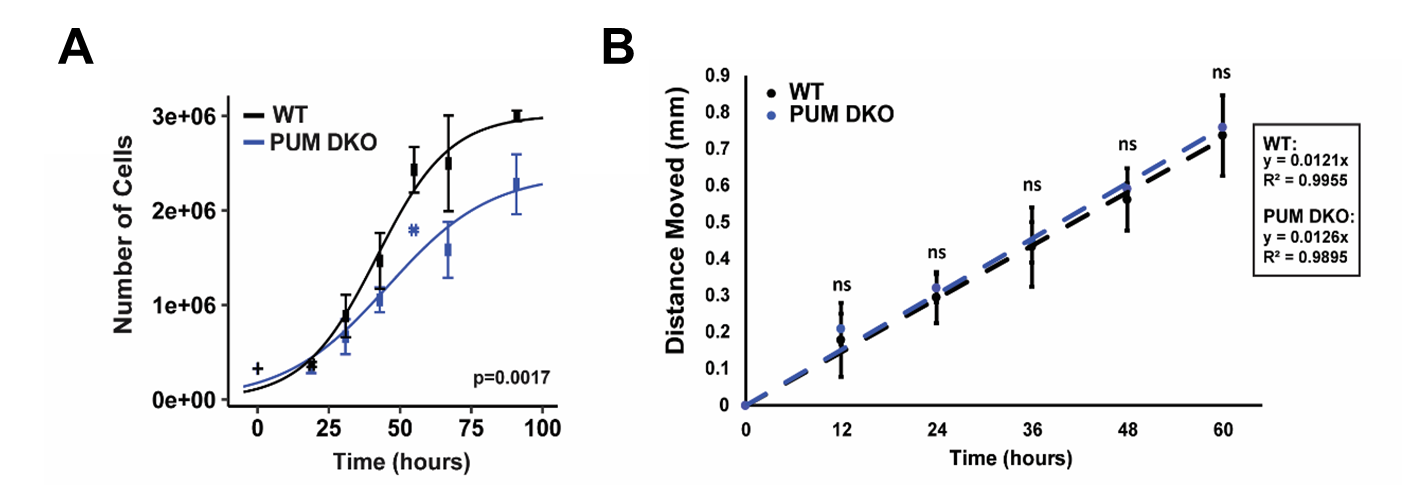


**Supplementary Figure 1: HCT116 PUM DKO cells show growth defects, but not strong adhesion and migration defects.** (A) Growth rates of WT (black) and PUM DKO (blue) HCT116 cells. Growth measurements were calculated from three biological replicates, with error bars representing standard error of the mean. For the purposes of plotting, the time points are grouped for each cell type / replicate group (since the data was collected at slightly different timepoints for each replicate), generating error bars along the x axis direction (standard error of the mean). Logistic growth model fits of the data were compared using ANOVA F-test to determine statistical significance. (B) Migration rates of WT (black) and PUM DKO (blue) cells. Measurements were calculated over 3 biological replicates. Statistical significance was determined by Student's T-test.


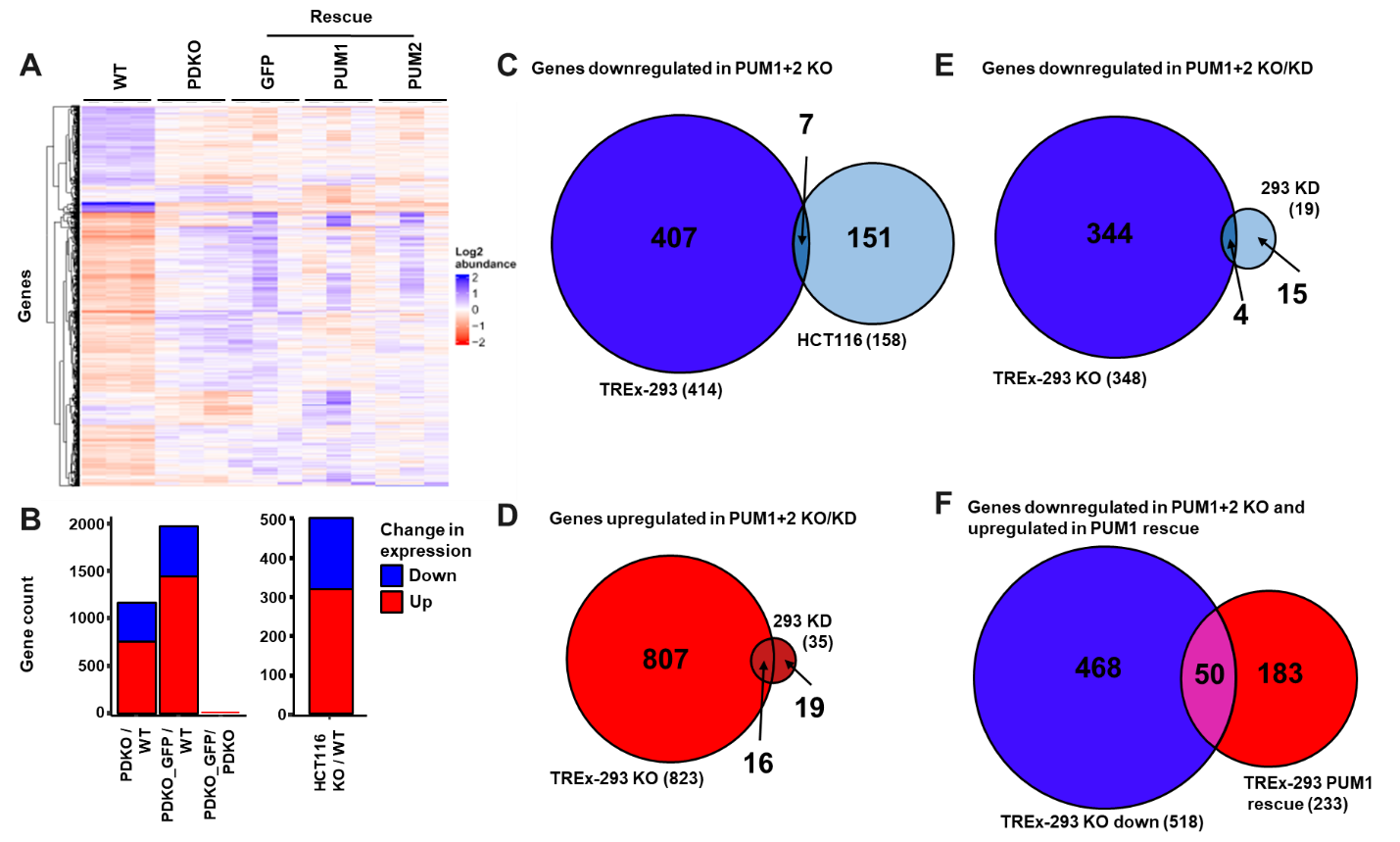


**Supplementary Figure 2:** (A) Heatmap of RNAseq gene expression changes between WT, PDKO and rescue T-REx-293 cells. (B) Number of differentially expressed genes in T-REx-293 PDKO vs WT, PDKO+GFP vs WT, and PDKO+GFP vs PDKO cells (left), and in HCT116 PDKO vs WT cells (right). (C) Overlap among genes downregulated in PDKO vs WT T-REx-293 and HCT116 cells. (D) Overlap among genes upregulated in PDKO vs WT T-REx-293 cells and 293 PUM1+2 KD cells. (E) Overlap among genes downregulated in PDKO vs WT T-REx-293 cells and 293 PUM1+2 KD cells. (F) Overlap of genes downregulated in T-REx-293 PDKO vs WT with genes upregulated in PUM1 rescue vs PDKO.

### **Supplementary Figure 2:** (A) Heatmap of RNAseq gene expression changes between WT, PDKO and rescue T-Rex-293 cells. (B) Number of differentially expressed genes in T-REx-293 PDKO vs WT, PDKO+GFP vs WT, and PDKO+GFP vs PDKO cells (left), and in HCT116 PDKO vs WT cells (right). (C) Overlap among genes downregulated in PDKO vs WT T-REx-293 and HCT116 cells. (D) Overlap among genes upregulated in PDKO vs WT T-REx-293 cells and 293 PUM1+2 KD cells. (E) Overlap among genes downregulated in PDKO vs WT T-REx-293 cells and 293 PUM1+2 KD cells. (F) Overlap of genes downregulated in T-Rex-293 PDKO vs WT with genes upregulated in PUM1 rescue vs PDKO.

**Supplementary Figure 3: PDKO Trex-293 cells are unable to form a monolayer typical of WT Trex-293 cells and this phenotype can be moderately alleviated by the exogenous expression of PUM1 or PUM2.** (A) WT (black) and PDKO (blue) T-REx-293 average number of space objects. (B). Stable integrant populations of PUM1 (green), PUM2 (red), or GFP (blue) in PDKO T-REx-293 cells were analyzed for average number of space objects. Measurements were calculated from six biological replicates. Shaded band represents two standard deviations from the mean. Statistical significance was determined by ANOVA F-test.

**Supplementary Figure 2:** (A) Heatmap of RNAseq gene expression changes between WT, PDKO and rescue T-Rex-293 cells. (B) Number of differentially expressed genes in T-REx-293 PDKO vs WT, PDKO+GFP vs WT, and PDKO+GFP vs PDKO cells (left), and in HCT116 PDKO vs WT cells (right). (C) Overlap among genes downregulated in PDKO vs WT T-REx-293 and HCT116 cells. (D) Overlap among genes upregulated in PDKO vs WT T-REx-293 cells and 293 PUM1+2 KD cells. (E) Overlap among genes downregulated in PDKO vs WT T-REx-293 cells and 293 PUM1+2 KD cells. (F) Overlap of genes downregulated in T-Rex-293 PDKO vs WT with genes upregulated in PUM1 rescue vs PDKO.


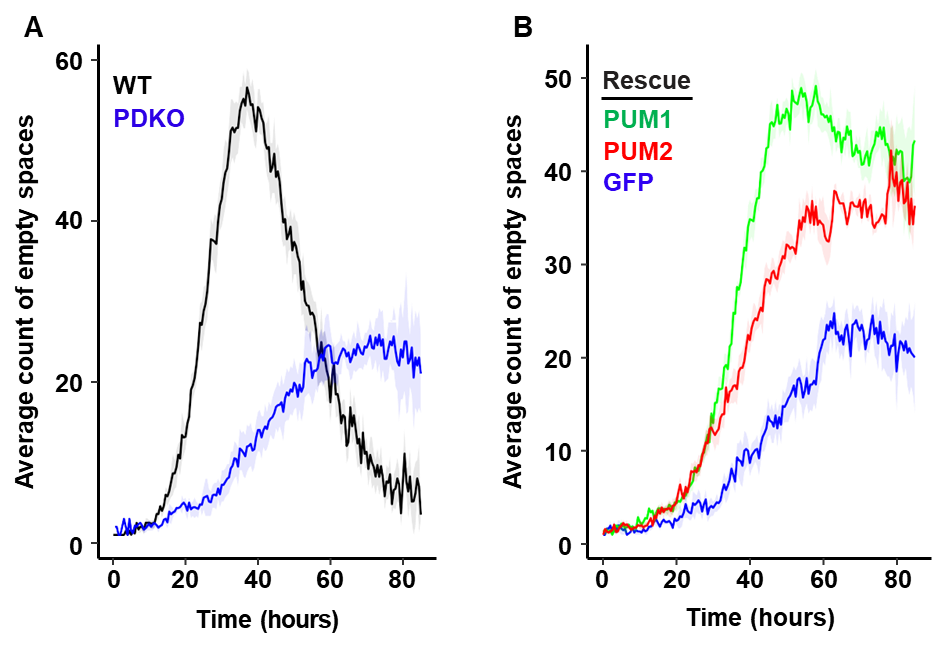


**Supplementary Figure 3: PDKO T-REx-293 cells are unable to form a monolayer typical of WT T-REx-293 cells and this phenotype can be alleviated by the exogenous expression of PUM1 or PUM2.** (A) WT (black) and PDKO (blue) T-REx-293 average number of space objects. (B). Stable integrant populations of PUM1 (green), PUM2 (red), or GFP (blue) in PDKO T-REx-293 cells were analyzed for average number of space objects. Measurements were calculated from six biological replicates. Shaded band represents two standard deviations from the mean.


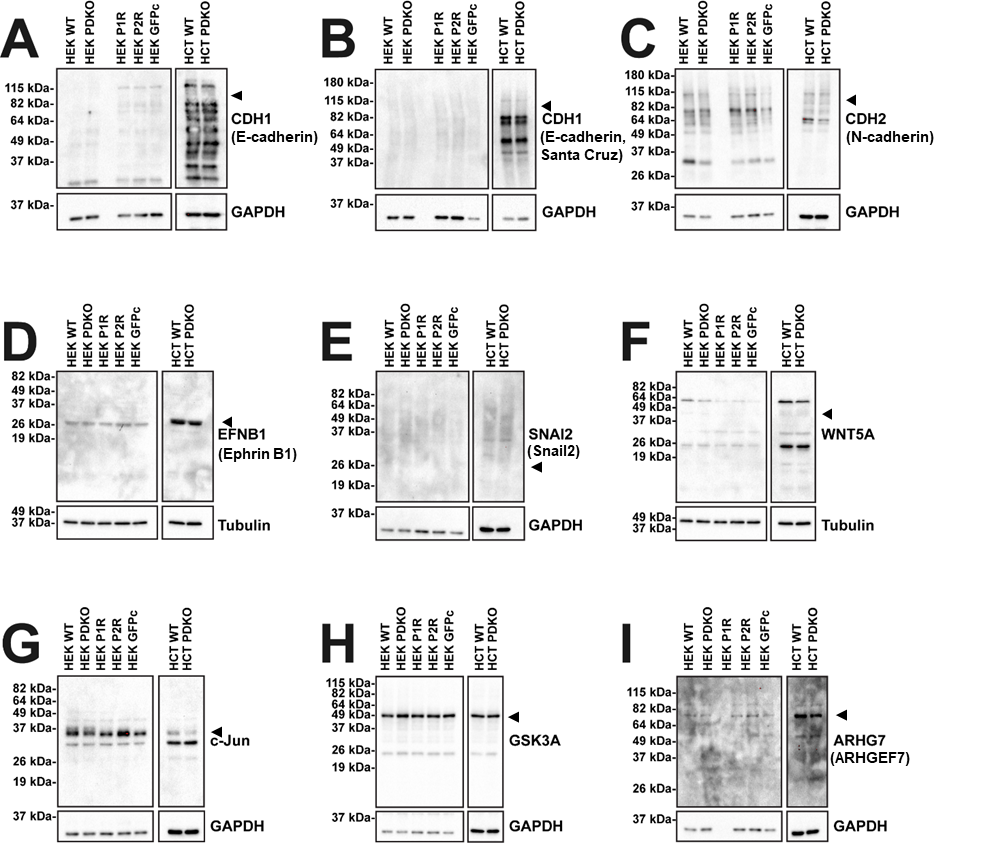


**Supplementary Figure 4: The PDKO adhesion phenotype is not caused by changes in cadherin expression levels, or levels of select candidate genes.** Western blots across WT, PDKO, and rescue T-REx-293 cells for (A) E-cadherin obtained from BD Transduction Laboratories, 610181 and (B) E-cadherin obtained from Santa Cruz, sc-8426 (C) N-cadherin, (D) Ephrin B1, (E) Snail2, (F) Wnt5A, (G) c-Jun, (H) GSK-3𝛼, and (I) Arhgef7. Arrowheads represent predicted band size based on molecular weight. Uncropped original blots are presented in Supplementary Figures 8-16.

*
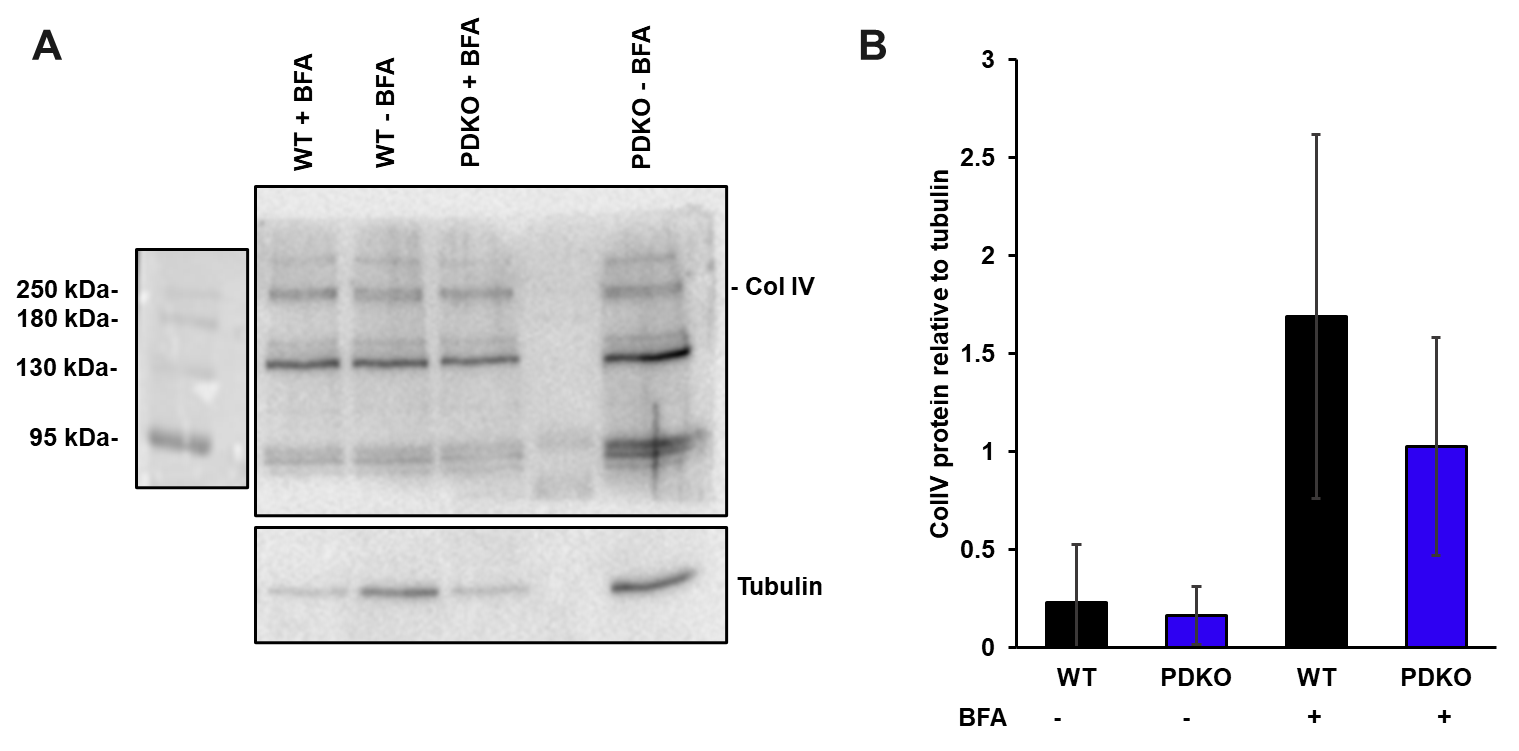
*

**Supplementary Figure 5: ColIV protein levels do not change upon PDKO.** (A) Representative western blot image of ColIV. Tubulin serves as a loading control. (B) Western blot quantification of ColIV protein compared to total protein in WT and PDKO T-REx-293 cells with and without the addition of BFA. Error bars represent standard deviation. Uncropped original blots are presented in Supplementary Figure 17.


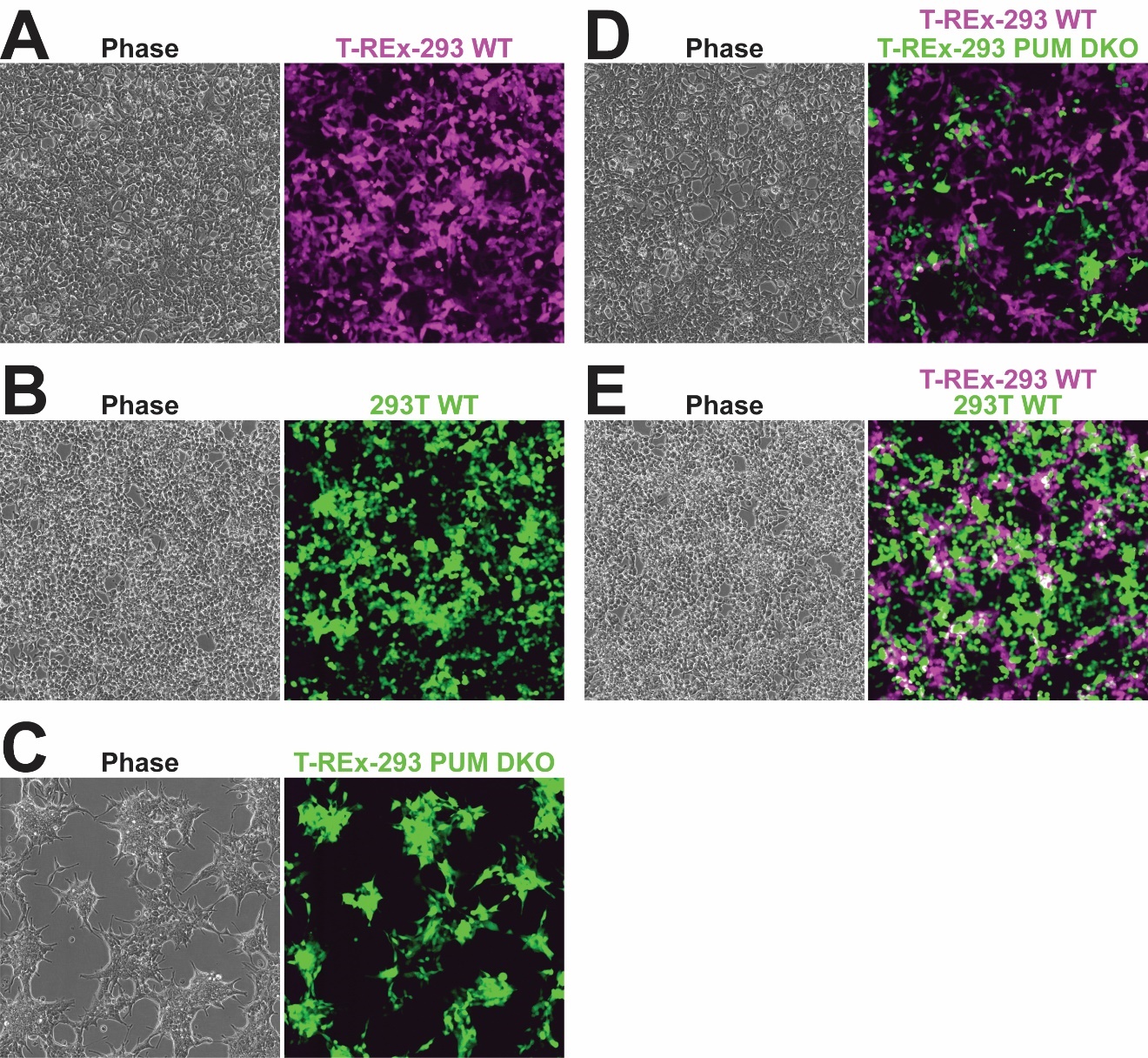


**Supplementary Figure 6: Co-culture of WT and PDKO cells rescues the increased cell adhesion phenotypes.** (A) Phase contrast and fluorescence images of T-REx-293 WT only, (B) 293T WT only, (C) T-REx-293 PUM DKO only, (D) co-cultured T-REx-293 WT and PUM DKO, and (E) co-cultured T-REx-293 WT and 293T WT cells. Images were collected after 72 hours of culture.


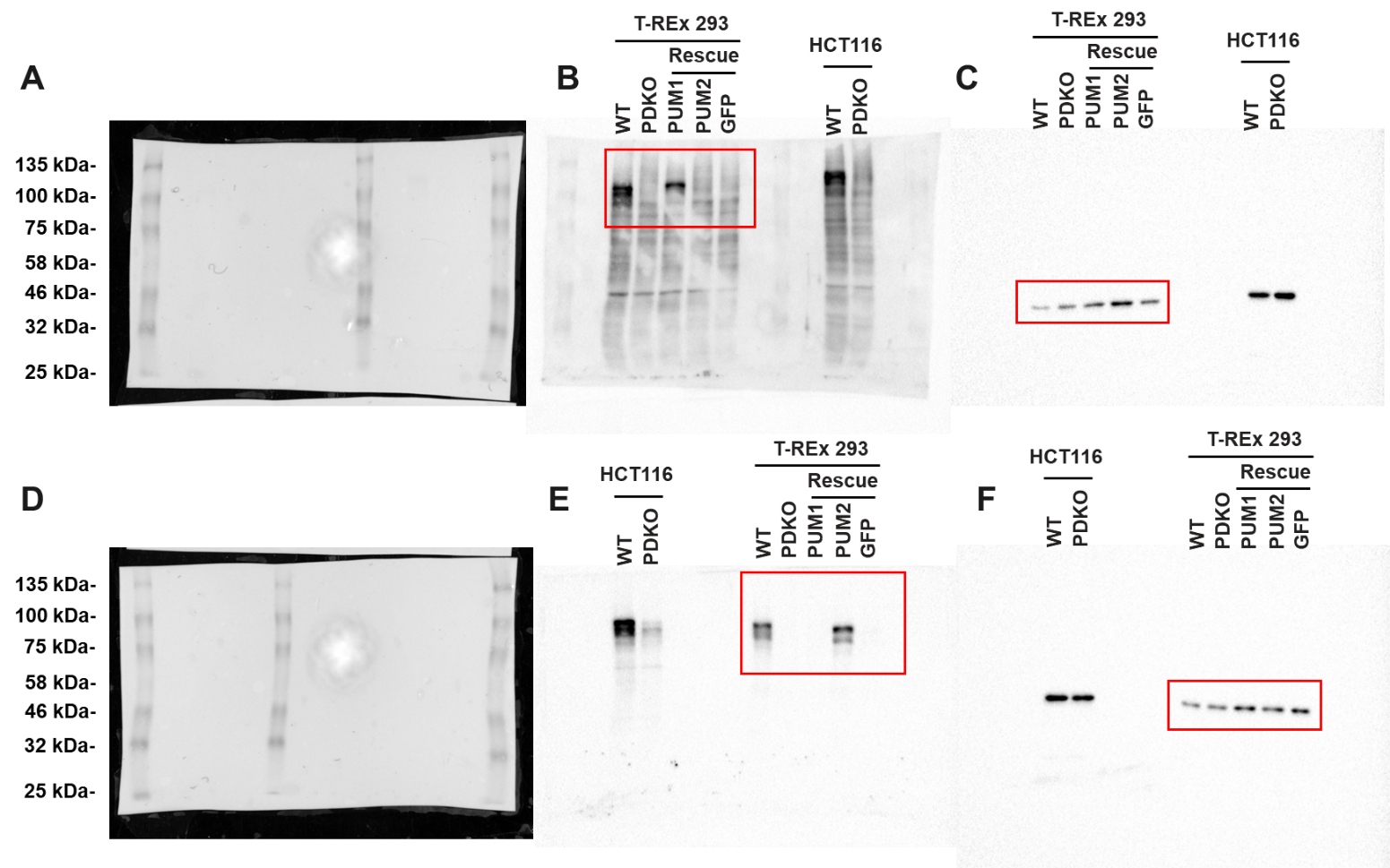


**Supplementary Figure 7:** Full length blots from Figure 1A. (A) Colorimetric image of PUM1 blot, (B) PUM1, and (C) Tubulin from PUM1 blot. (D) Colorimetric image of PUM2 blot, (E) PUM2, and (F) Tubulin from PUM2 blot. Red boxes represent the cropped images used in Figure 1A.


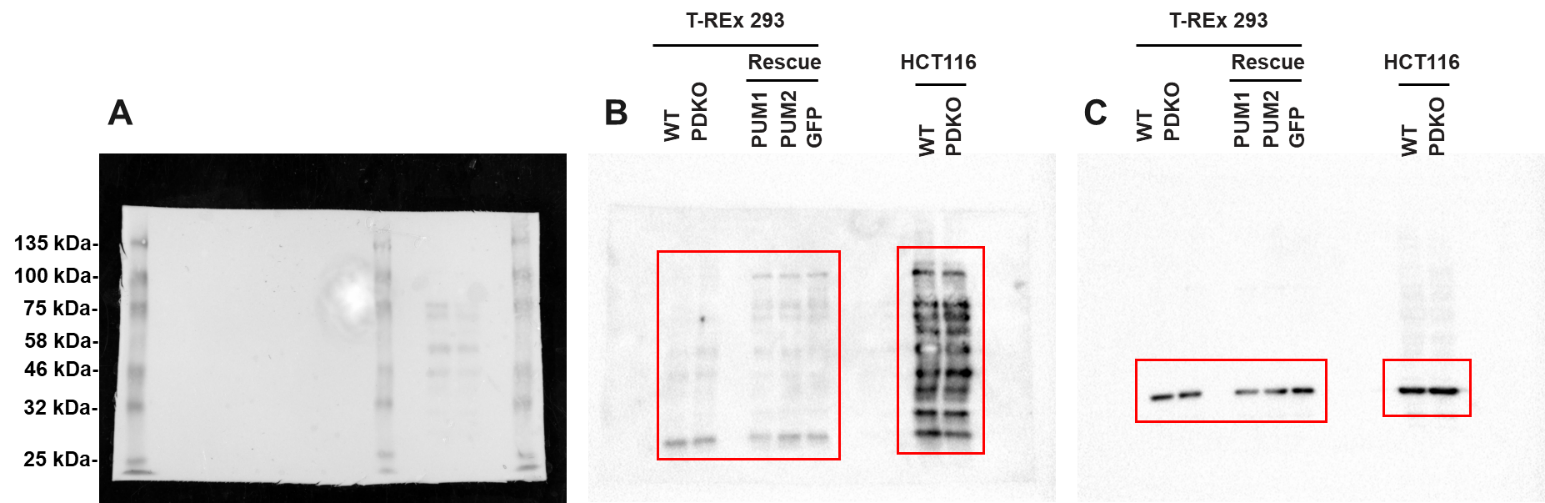


Supplementary Figure 8: Full length blot of E-cadherin from Supplementary figure 4A. Antibody purchased from BD Transduction Laboratories, 610181. (A) Colorimetric image, (B) CDH1, (C) GAPDH. Red boxes represent the cropped images used in Supplementary figure 4A.


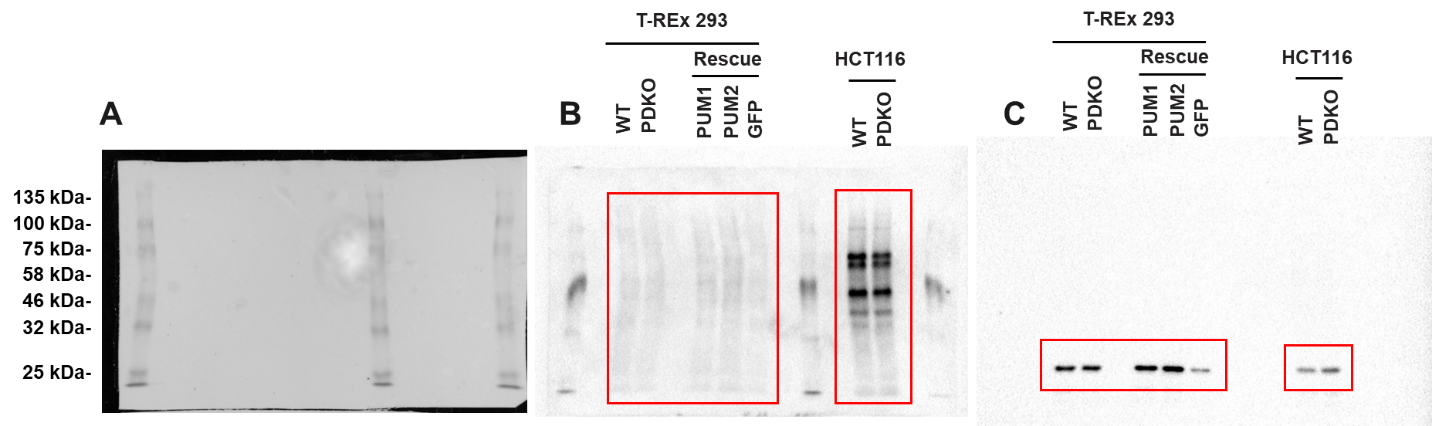


Supplementary Figure 9: Full length blot of E-cadherin from Supplementary figure 4B. Antibody purchased from Santa Cruz, sc-8426. (A) Colorimetric image, (B) CDH1, (C) GAPDH. Red boxes represent the cropped images used in Supplementary figure 4B.


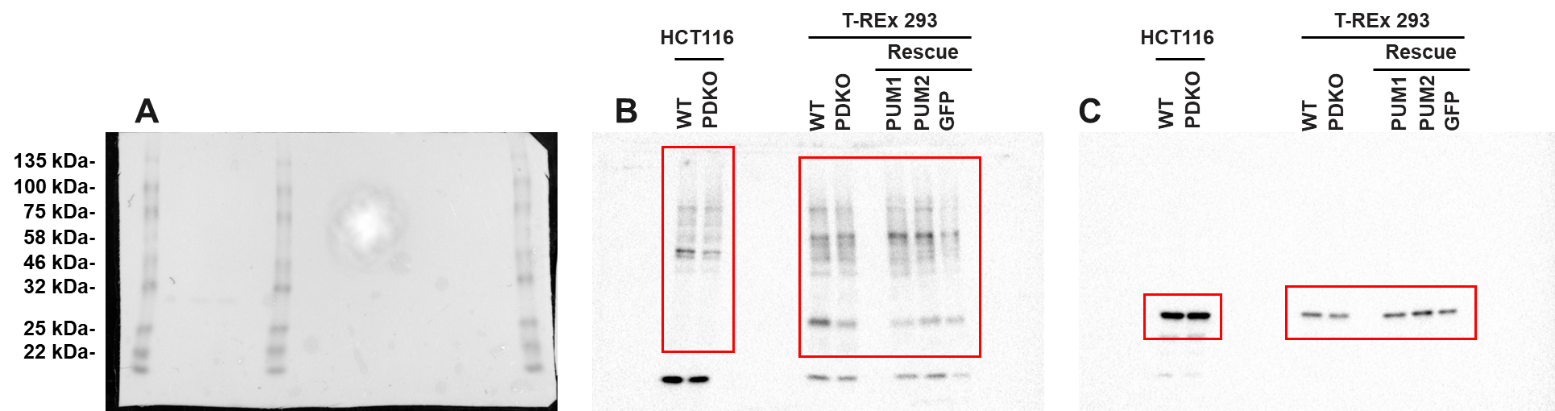


Supplementary Figure 10: Full length blot of N-cadherin from Supplementary figure 4C. (A) Colorimetric image, (B) CDH2, (C) GAPDH. Red boxes represent the cropped images used in Supplementary figure 4C.


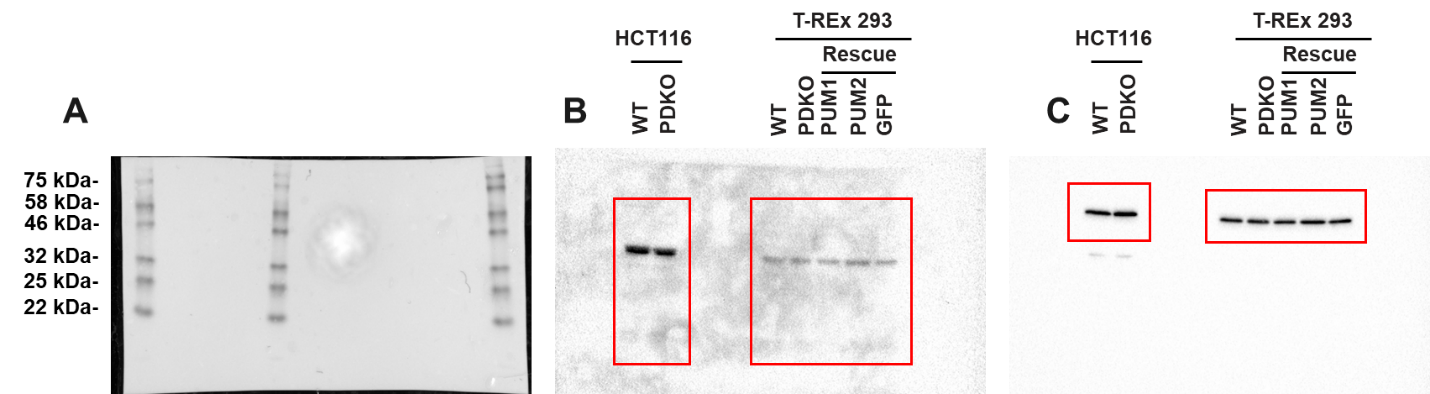


Supplementary Figure 11: Full length blot of Ephrin B1 from Supplementary figure 4D. (A) Colorimetric image, (B) EFNB1, (C) Tubulin. Red boxes represent the cropped images used in Supplementary figure 4D.


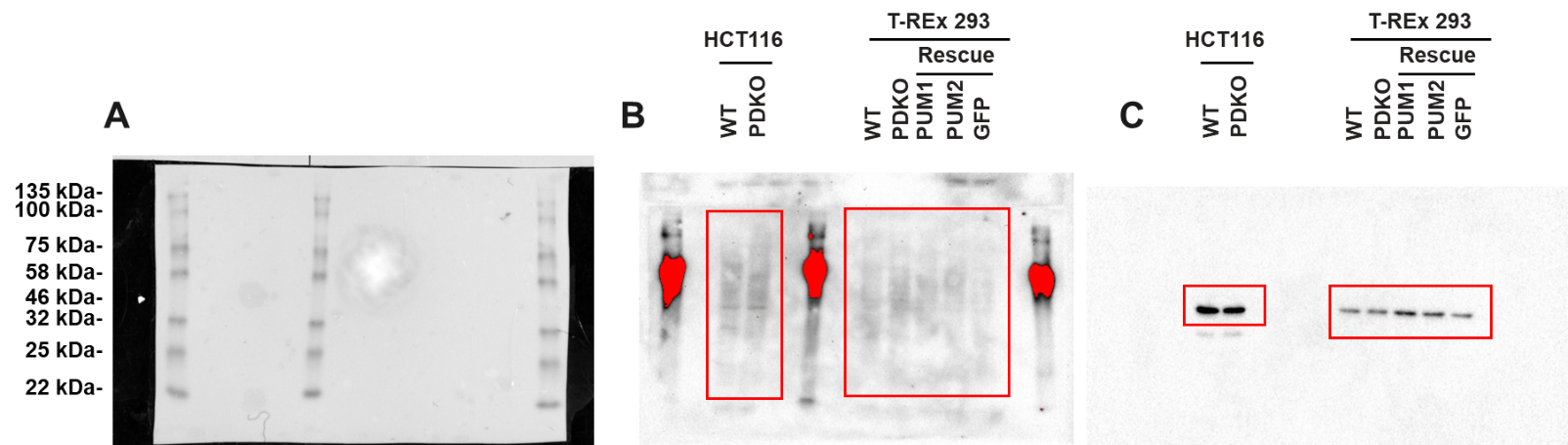


Supplementary Figure 12: Full length blot of Snai2 from Supplementary figure 4E. (A) Colorimetric image, (B) Snai2 (Bands show cross reactivity with 75kDa band on ladder), (C) GAPDH. Red boxes represent the cropped images used in Supplementary figure 4E.


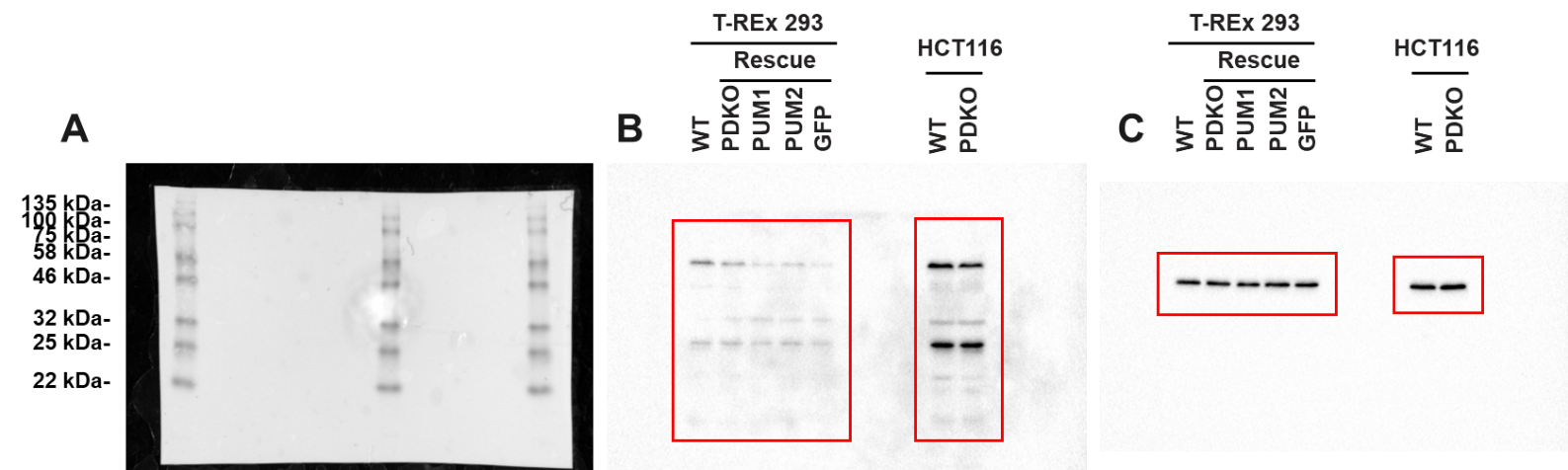


Supplementary Figure 13: Full length blot of Wnt5a from Supplementary figure 4F. (A) Colorimetric image, (B) Wnt5a (C) Tubulin. Red boxes represent the cropped images used in Supplementary figure 4F.


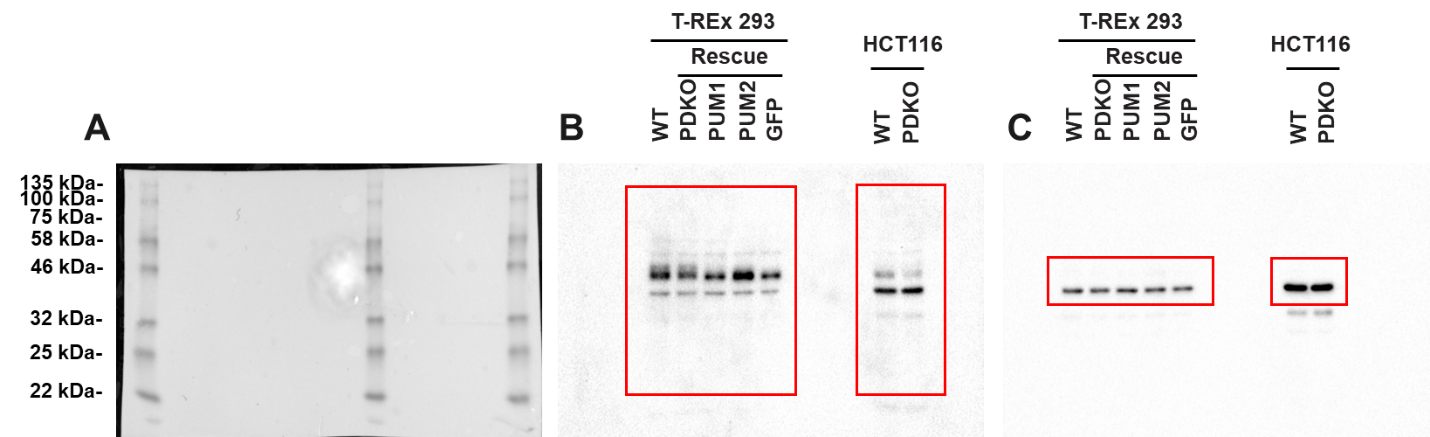


Supplementary Figure 14: Full length blot of C-Jun from Supplementary figure 4G. (A) Colorimetric image, (B) C-Jun (C) Tubulin. Red boxes represent the cropped images used in Supplementary figure 4G.


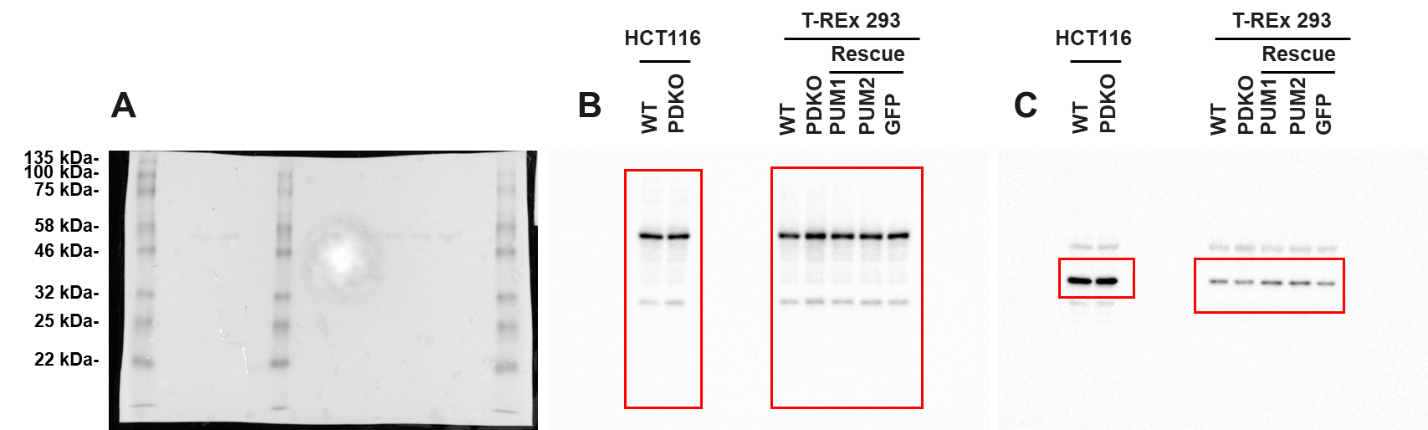


Supplementary Figure 15: Full length blot of GSK3A from Supplementary figure 4H. (A) Colorimetric image, (B) GSK3A (C) Tubulin. Red boxes represent the cropped images used in Supplementary figure 4H.


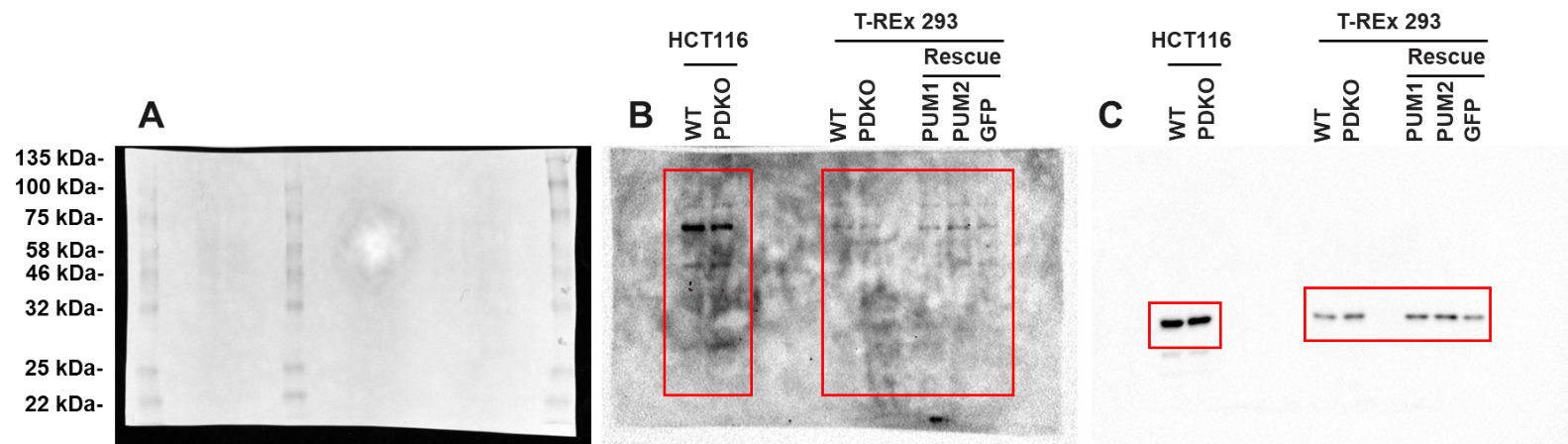


Supplementary Figure 16: Full length blot of ARHG7 (bpix) from Supplementary figure 4I. (A) Colorimetric image, (B) ARHG7 (C) GAPDH. Red boxes represent the cropped images used in Supplementary figure 4I.


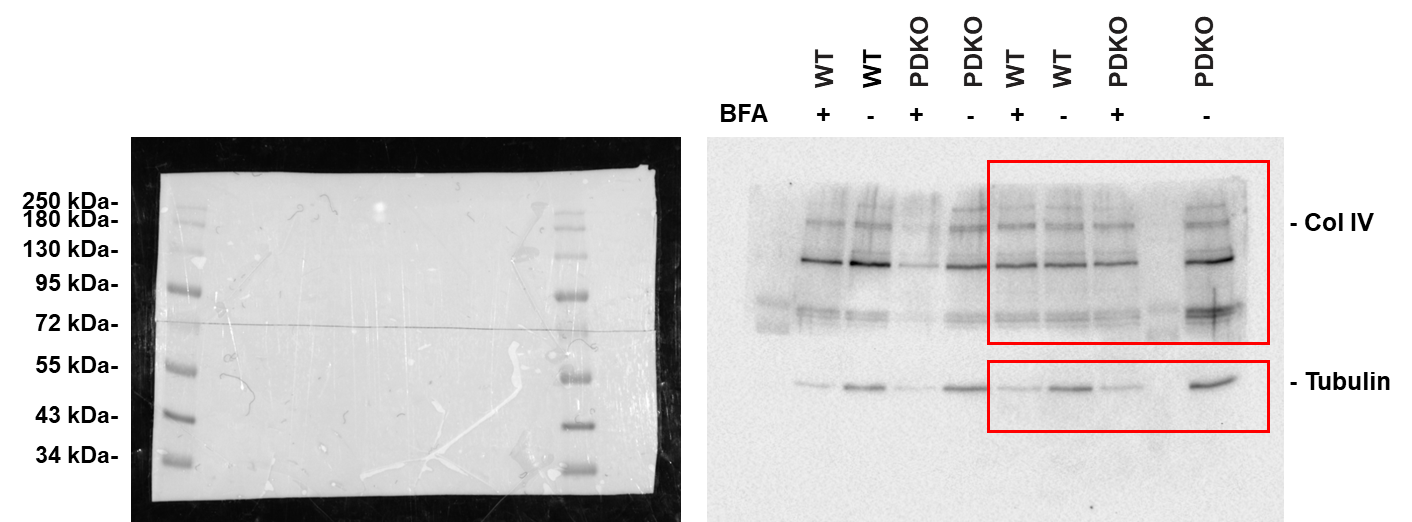


Supplementary Figure 17: Full length blot of Collagen IV, with and without the use of BFA, from Supplementary figure 5A. (A) Colorimetric image, (B) immunoblot for ColIV and Tubulin. Red boxes represent the cropped images used in Supplementary figure 5.

## Supplementary Video Legends:

**Supplementary Video 1: Time-lapse video of WT T-REx-293 cells.** Frames correspond to 15-minute intervals over a 72-hour period.

**Supplementary Video 2: Time-lapse video of PUM DKO T-REx-293 cells.** Frames correspond to 15-minute intervals over a 72-hour period.

**Supplementary Video 3: Time-lapse video of PUM DKO T-REx-293 cells stably expressing GFP.** Frames correspond to 15-minute intervals over a 72-hour period.

**Supplementary Video 4: Time-lapse video of PUM DKO T-REx-293 cells stably expressing PUM1.** Frames correspond to 15-minute intervals over a 72-hour period.

**Supplementary Video 5: Time-lapse video of PUM DKO T-REx-293 cells stably expressing PUM2.** Frames correspond to 15-minute intervals over a 72-hour period.
